# NutricheQ Questionnaire assesses the risk of dietary imbalances in toddlers from 1 through 3 years of age

**DOI:** 10.3402/fnr.v59.29686

**Published:** 2015-12-18

**Authors:** Giuseppe S. Morino, Giulia Cinelli, Ilaria Di Pietro, Vittoria Papa, Nicola Spreghini, Melania Manco

**Affiliations:** Research Area for Multifactorial Diseases and Nutritional Department, Bambino Gesù Children's Hospital, Rome, Italy

**Keywords:** preschooler, diet, dietary deficiency, micronutrient intake, nutritional questionnaire, iron, vitamin D deficiency

## Abstract

**Background:**

Although a nutrient-poor diet may affect children's growth, especially early in life, few tools to assess dietary imbalances in 1- to 3-year-old children have been developed.

**Objectives:**

To investigate the accuracy and test–retest reliability of the NutricheQ Questionnaire in the identification of toddlers with the risk of inadequate intake of micro- and macronutrients in a sample of Italian toddlers.

**Design:**

A 3-day weighed food record was performed, and results were compared with outcomes of the NutricheQ Questionnaire in 201 toddlers (training set: 1–3 years old). The accuracy of NutricheQ in the identification of categories of nutritional risk was evaluated using the receiver operating characteristic (ROC) curves. Test–retest of the tool was estimated using the intraclass correlation coefficient (ICC) and the Cronbach's alpha statistic, in a validation set of 50 toddlers.

**Results:**

The NutricheQ Questionnaire is a valid tool for the identification of toddlers at risk for dietary imbalances. Significant differences in nutrient intake (*p*<0.005) were found among the three groups of risk identified by the questionnaire: toddlers included in the high-risk group had a lower intake of key nutrients such as iron, vitamin D and other vitamins, and fibre compared to those included in the low-risk group. NutricheQ is also reliable between administrations, as demonstrated by its test–retest reliability. ICC and Cronbach's alpha were 0.73 and 0.83, respectively, for Section 1 of NutricheQ, and 0.55 and 0.70 for Section 2.

**Conclusions:**

The NutricheQ Questionnaire is a reliable and consistent tool for the assessment of possible dietary risk factors in Italian toddlers. It consistently identifies toddlers with a high probability of having poor iron and vitamin D intake, and other dietary imbalances.

Inadequate intakes of both macronutrients (i.e. proteins, fats, and carbohydrates) (1–3) and micronutrients, such as iron and vitamin D (4), are highly prevalent among toddlers in westernised countries. The European Food Safety Authority (EFSA) Panel on Dietetic Products, Nutrition and Allergies (NDA) acknowledged 21 publications reporting on nutrient intakes in European toddlers. Energy and protein intakes were in all surveys excessive, being above the average requirement (AR) or the population reference intake (PRI). Iron and vitamin D intakes, on the contrary, were generally below the PRI or AR, resulting in a prevalence of deficiency varying around 1–20% for iron and 10–30% for vitamin D (5).

In the United States, a similar trend has been observed. The latest exhaustive survey of dietary patterns in infants, from the Feeding Infants and Toddlers Study (FITS) in North America, reports current trends in infant feeding with excessive intake of proteins and fats and poor consumption of fruits and vegetables, plus high levels of starchy, rather than green or yellow, vegetables (2). The excess in proteins intake has been associated with an enhanced risk of developing obesity and cardiometabolic abnormalities in childhood and early adulthood (6, 7).

A widespread feeding habit that might lead to dietary imbalances is the excessive consumption of cow's milk. Most countries recommend the delayed introduction of cow's milk, until infants are at least 12 months in age, even if small volumes are added to complementary foods (8). The main reason is that cow's milk is a poor source of iron: daily consumption above 500 ml and the resulting avoidance or substitution of other iron-rich foods might promote iron deficiency with or without overt anaemia (9). Iron deficiency impairs the growing child's neurodevelopment, learning, and memory capacities (10, 11). Above all, cow's milk contains a higher amount of proteins, compared to infant formula, which seems to have a specific effect on growth through the modulation of insulin-like growth factor-1 (IGF-1) concentration. Evidence from the literature suggests that high protein intake during the first two years of life is a risk factor for the later development of overweight and obesity. Therefore, excessive consumption of cow's milk, especially as a main drink, might have negative outcomes in later stages of life, inducing a higher growth rate and increasing the risk of later overweight and obesity (6).

Dietary imbalance that precedes rapid growth at this age results also in mild vitamin D deficiency. The latter may be associated with several adverse health outcomes in toddlers, including an increased prevalence of infectious diseases (viral upper respiratory tract infections), allergic diseases, type 1 diabetes mellitus, or cardiovascular diseases (12, 13).

Prompt recognition of inadequate intakes is advisable and attention should be paid to complementary foods in order to prevent nutritional imbalances (8).

Hence, there is a need for reliable age-specific nutritional tools, designed for the identification of toddlers at risk for dietary imbalances, who might need healthcare support or even therapeutic intervention.

There are some standardised tools (e.g. the Toddlers Eating Behaviour Questionnaire, and the Dutch Eating Behaviour Questionnaire for Toddlers) that aim at investigating eating habits associated with the development of eating disorders, such as restrained, emotional, and external eating (14, 15). Unfortunately, they do not provide any information about the intake of macro- and micronutrients. On the contrary, the NutriSTEP, ‘Nutrition Screening Tool for Every Preschooler’, was developed to evaluate the nutritional risk associated with an inadequate intake of macro- and micronutrients, but in preschoolers (3- to 5-year-old toddlers). It includes 17 items referring to the consumption of different food categories and eating behaviours (16). Recently, a new dietary questionnaire, the Toddler Dietary Questionnaire (TDQ), has been developed and validated in toddlers (aged 1–3 years). The tool identifies four risk categories (low, moderate, high, and very high) in Australians, depending on the score obtained (17).

Very recently, Danone Nutricia Early Life Nutrition developed the NutricheQ Questionnaire as a tool for family paediatricians, with the objective of assessing dietary risk factors in 1- to 3-year-old children. It identifies three categories of nutritional risk (high, moderate, and low), which are based on the reduced consumption of some micronutrients (mainly iron and vitamin D) and the excessive intake of foods that are energy-dense, rich in proteins and saturated fatty acids, but micronutrient-poor. The aim is to identify toddlers with high probability of having nutrients imbalances, who might need nutritional intervention. It has also been thought of as a means to help parents choose the correct eating habits for their children, ensuring that adequate nutritional intakes are met.

The objectives of the present study were: (i) to evaluate the accuracy of the NutricheQ Questionnaire in the identification of those toddlers with possible inadequate intake of some nutrients, in a training set of 1- to 3-year-old Italian toddlers and (ii) to evaluate its test–retest reliability in a validation set of 50 toddlers.

## Methods

### Participants

Toddlers (1–3 years old) were enrolled by 13 family paediatricians in four metropolitan areas surrounding Rome (Rome, Latina, Frosinone, Caserta). Each paediatrician enrolled 24–26 toddlers (50% males), providing a total sample of 320 participants whose parents agreed to be included in the study. Exclusion criteria were chronic, gastric or intestinal diseases, and food allergies, that might influence appetite or eating behaviours. Because of the high number of dropouts expected, beginning with 320 toddlers (160 males) ensured at least 200 participants.

The survey was approved by the Ethical Committee at the Bambino Gesù Children's Hospital.

### Anthropometrics

The paediatrician enrolled participants in periodic visits for growth monitoring. After informing parents about the aim of the research and obtaining their written informed consent, the paediatrician measured the child's weight and length. Anthropometric measurements were performed according to standardised procedures (18). The mean of two measurements was used. Weight/age, length/age, and weight/length ratio percentiles were assessed using the World Health Organization (WHO) curve standards (Anthro software program for Windows, version 3.2.2) (19). According to the WHO growth standards, toddlers with weight/length ratio below the 3rd or above the 97th percentile were classified as underweight and overweight, respectively. Toddlers were considered at risk of malnutrition if below the 10th percentile or at risk of overweight if above the 90th percentile (20, 21).

### The NutricheQ Questionnaire

Three registered nutritionists (GC, IDP, and VP) instructed mothers by phone to fill the electronic NutricheQ Questionnaire that was sent via e-mail with written instructions and a standardised questionnaire on the Socio-Economical Status (SES). The questionnaire was returned by e-mail.

The NutricheQ Questionnaire has been developed by Danone Nutricia Early Life Nutrition based on suggestions from family paediatricians, the user-requirements of European nutrition experts, and evidence from state-of-the-art literature (22). NutricheQ results in a two-section, 11-item questionnaire that takes between 3 and 5 minutes to complete. Section 1 was designed primarily to identify dietary risk factors that could lead to low iron and vitamin D intake, (Question 1–4); Section 2 focuses on other dietary imbalances related to an excessive consumption of energy-dense, micronutrient-poor foods and drinks, and fewer fruits and vegetables (Question 5–11). NutricheQ focuses on those aspects of the toddler's diet that are markers for inadequate or excessive intake/dietary imbalances. To make the questionnaire as easy as possible to complete and score within a clinical setting, the number of possible responses per question was limited to three (a, b, c). Answers in the ‘a’ category are considered appropriate or desirable (score of 0); ‘b’ category less than ideal (score of 1); and ‘c’ category indicating a potential cause for concern/action (score of 2). By adding scores within each section, an indicator of risk was obtained for the aspect of the toddler's diet that the section is designed to evaluate, whereas the combined scores for Sections 1 and 2 give an overall rating based on all aspects of dietary intake. The maximum total score obtainable was, therefore, 22 from Sections 1 and 2.

The NutricheQ Questionnaire provides a semi-quantitative estimation of daily milk consumption; and the frequency of consumption of red meat, oily and dark fish, cereals fortified with iron and vitamins, fruit, vegetables, dairy products, ‘convenience/fast’ foods, and fruit juices or sweetened drinks. Table 1 summarises the food categories investigated in Sections 1 and 2, and the score-derived risk categories. Supplementary Table 1 shows the Italian version of the NutricheQ Questionnaire.

**Table 1:** Brief description of the NutricheQ Sections 1 and 2 and meaning of the associated scores

Sections	Investigated Foods	Items (N)	Score
Section 1	Milk consumption; breakfast products, meat, fish.	4	*0–1: Low risk* Low probability of a poor intake of micronutrients such as iron, vitamin D, and zinc. *2–3: Moderate risk* Moderate probability of a poor intake of micronutrients such as iron, vitamin D, and zinc. ≥*4: High risk* High probability of a poor intake of micronutrients such as iron, vitamin D, and zinc.
Section 2	Processed foods, confectionary, non-milk beverages, fruits, and vegetables	7	*0–2: Low risk* Low probability of an excessive intake of saturated fatty acids, sugars, and sodium. *3–5: Moderate risk* Moderate probability of an excessive intake of such as saturated fatty acids, sugars, and sodium. ≥*6: High risk* High probability of an excessive intake of saturated fatty acids, sugars, and sodium.

### Food consumption assessment and estimation of nutrient intakes

A 3-day weighed food record (3DWFR) was used to register food and beverage intake data. Mothers were asked to record the child's food consumption during a 3-weekday period. They did not receive any specific nutritional recommendations prior to the study, so that each child's dietary recall reflected the daily food consumption according to the parents’ choices. Registered nutritionists instructed parents after they completed the questionnaire and gave support when needed. The 3DWFR was sent back within a week. The quantity, type, and brand of all foods and beverages were recorded. Parents were asked to specify the food amount using measurement units (e.g. grams, millilitres), packaging size or household measures (e.g. cup, teaspoon, or tablespoon). The ‘LARN (*Livelli di Assunzione di Riferimento di Nutrienti ed energia per la popolazione italiana) Standard delle Porzioni* 2014’ tables were used to assure a standardised evaluation of food amount, if reported in household measures (i.e. according to LARN *Standard delle Porzioni*, a teaspoon contains 5 g of extra virgin olive oil, while a tablespoon contains 9 g) (23). The average intake of nutrients was estimated using specific nutritional software (DietoSystem DS Medica S.r.l., Milano, Italy) and compared to the Italian dietary reference values (DRVs) (LARN), specific for toddlers (Table 2).

**Table 2:** Energy and nutrient intakes in the study sample (*n*=201) obtained from the 3-day weighed food record

Nutrients^a^	Median (IQR)	AR	AI	RI	SDT
Energy (kJ)	Males: 4539.6 (1234.3) Females: 4234.2 (983.2)	M: 3640.1–5815.8 F: 3305.4–5355.5			
Proteins (g)	44.3 (15.4)				
Proteins (g/kg BW)	3.3 (1.3)	0.82			
Proteins (%TE)	16.5 (3.6)				<15
Carbohydrates (g)	131.8 (43.4)				
Carbohydrates (%TE)	47.8 (6.7)			45–60	
Sugars (g)	52.6 (22.9)				
Sugars (%TE)	19.2 (7)				
Fats (g)	39.9 (12.6)				
Fats (%TE)	34.8 (5.7)			35–40	
Saturated fatty acids (g)	12.9 (6.6)				
Saturated fatty acids (%TE)	10.9 (4.1)				<10
Total fibre (g)	7.6 (4.3)				
Fibre (g/4184kJ)	7.1 (3.7)		8.4		
Thiamine (mg)	0.6 (0.2)	0.3			
Riboflavin (mg)	1.1 (0.5)	0.4			
Niacin (mg NE)	7.6 (3.9)	5			
Folic acid (µg)	131.9 (77.1)	110			
Vitamin A (µg RE)	573.3 (426.8)	200			
Vitamin C (mg)	63.64 (55.2)	25			
Vitamin D (µg)	0.98 (1.8)	10			
Vitamin E (mg)	6.7 (2.4)		5		
Sodium (mg)	649.3 (373.2)		700		900
Potassium (mg)	1494.5 (630)		1700		
Iron (mg)	5.7 (2.4)	4			
Calcium (mg)	608.3 (305)	500			
Phosphorum (mg)	759.4 (282)	380			
Zinc (mg)	5.9 (2.5)	4			
Selenium (µg)	22.4 (14.8)	16			
Fruits (g)	100 (117)				
Vegetables (g)	20 (52)				
				
**Nutrients^b^**					
Energy (kJ)	*N* (M)=11 (10.2%)				
	*N* (F)=8 (8.6%)				
Proteins (%TE)	*N*=140 (69.7%)				
Carbohydrates (%TE)	*N*=5 (2.5%)				
Sugars (%TE)	*N*=159 (79.1%)				
Fats (%TE)	*N*=28 (13.9%)				
Saturated fatty acids (%TE)	*N*=124 (61.7%)				
				
**Nutrients^c^**	
Fibre (g/4184kJ)	*N*=129 (64.2%)				
Thiamine (mg)	*N*=2 (1.0%)				
Riboflavin (mg)	*N*=1 (0.5%)				
Niacin (mg NE)	*N*=34 (16.9%)				
Folic acid (µg)	*N*=58 (28.9%)				
Vitamin A (µg RE)	*N*=8 (3.4%)				
Vitamin C (mg)	*N*=22 (11%)				
Vitamin D (µg)	*N*=201 (100%)				
Vitamin E (mg)	*N*=29 (14.4%)				
Sodium (mg)	*N*=112 (55.7%)				
Potassium (mg)	*N*=145 (72.1%)				
Iron (mg)	*N*=26 (12.9%)				
Calcium (mg)	*N*=61 (30.3%)				
Phosphorum (mg)	*N*=8 (4%)				
Zinc (mg)	*N*=21 (10.5%)				
Selenium (µg)	*N*=49 (24.4%)				

a Reference values for 1- to 3-year-old Italian toddlers are reported (12).

b The number and percentage of toddlers exceeding LARN recommendations for carbohydrates, fats, proteins, sugars, and saturated fatty acids intake.

c The number and percentage of toddlers who did not meet the LARN recommendations for vitamins and minerals.

Data are reported as median (IQR). 3DWFR: 3-day weighed food record. Reference values are reported as appropriate according to the LARN recommendations for 1- to 3-year-old toddlers (23). *Reference intake* (RI) is the recommended range for carbohydrates and fats as a percentage of total energy (TE) intake. *Average requirement* (AR) is the level of nutrient intake that satisfies 50% of the food requirement in a healthy population. *Adequate intake* (AI) indicates the recommended intake of fibres, vitamin E, sodium, and potassium. *Suggested dietary target* (SDT) is the suggested target (for quality and/or amount) intake for sugars and saturated fatty acids that is associated to a low risk of chronic degenerative diseases.

The intake of different carbohydrates, proteins, and fats; energy; fibre, fruit, and vegetables; vitamins B (B_1_, B_2_, B_3_), vitamin C, vitamin D, vitamin E, vitamin A and folic acid; minerals (sodium, potassium, calcium, phosphate, iron, zinc, selenium) were computed. Food consumption and nutrient intake were separately assessed by GC and IDP; in cases of inconsistencies, checks and comparisons were made until agreement was reached. Nutrient intakes were calculated and compared to the DRVs for the 1- to 3-year-old Italian population (23). An excessive intake of proteins was defined as an intake higher than the 15% of the total energy (TE) intake. Since the subjects included in the present study were totally recruited in Italy, LARN recommendations were considered appropriate to assess the adequacy of nutrient intakes; however, the EFSA scientific opinion on nutrient requirements for infants and toddlers throughout the European Union was taken into consideration. An EFSA opinion on DRVs is available for most of the nutrients that we considered (carbohydrates, proteins, and fats; fibre (g/4184 kJ); niacin, folic acid, and vitamins A and C; zinc and selenium). For other micronutrients, EFSA did not set DRVs, but reviewed the reference values provided by other scientific or authoritative bodies, providing advice on the levels of nutrients that are considered adequate for healthy, term, normal-weight toddlers (5). The DRVs or adequate levels for each nutrient are reported in Supplementary Table 2.

Significant differences in nutrients intake (3DWFR) between the three risk categories was assessed separately for Sections 1 and 2.

### Statistical analysis

Data are shown as median and inter-quartile range (IQR). The Mann-Whitney U test or The Kruskal–Wallis one-way analysis of variance (non-parametric analysis of variance by ranks) were used for the comparisons among the different groups of risk (low-, moderate-, and high-risk) derived from the NutricheQ scoring.

Receiver operating characteristic (ROC) curves were used to verify the accuracy of NutricheQ risk categories to identify toddlers with inadequate intake of certain nutrients according to the LARN recommendation. The method of maximum distance from the quarter's bisector (Youden's method) or the method of the minimum distance with the above corner on the left part of the quarter were used to identify appropriate risk cut points by comparing trade-offs of sensitivity and specificity indexes. An area under the ROC curve AUC=0.5 shows a not informative test; 0.5<AUC 0.7 is a not accurate test; 0.7<AUC 0.9 is a fairly accurate test; 0.9<AUC<1 is a highly accurate test; AUC=1 is a perfect test. Test–retest of the NutricheQ Questionnaire was estimated by the intraclass correlation coefficient (ICC) and the Cronbach's alpha statistic. A *p*-value of <5% was considered significant. Data analysis was performed using the R for Windows statistical software, version 3.0.3.

### Test–retest

In an additional sample of 50 subjects (25 males; validation set), the NutricheQ was completed twice within an interval of time ranging from 10 to 15 days. Both questionnaires were administered and returned by e-mail as described above and completed by the mothers.

## Results

### Sample description

Of the whole validation sample (*n*=320), 119 (37.2%) toddlers were excluded from the survey: 57 did not return the electronic questionnaire and 62 returned incomplete papers.

No difference was noticeable in the age, sex, and anthropometrics of those who completed the survey and those who did not (data not reported). There were no differences between the training set and the validation set, used to assess test–retest reliability.

Two-hundred and one toddlers (62.8%) were included in the survey (108 males, 53.7%). Median age was 26 (12) months, weight 12.8 (2.8) kg, and length 88 (9) cm. Three (1.5%) toddlers were underweight; 23 (1.4%) overweight; 15 (5.5%) at risk of under-nutrition; and 42 (20.9%) at risk of being overweight, according to WHO growth standards. Supplementary Table 3 reports detailed information on the sample SES.

### Comparison between NutricheQ risk categories and nutritional assessment

After the inclusion of each child into one of the risk groups, according to the NutricheQ score obtained, nutrients intake derived from the 3DWFR was taken into account: the median intake and IQR of each nutrient were separately calculated for low-, moderate-, and high-risk group. Then, statistical analysis was performed to assess any between-group significant difference in macro- and micronutrients intake (Table 3). Sections 1 and 2 were designed to investigate the adequacy of different nutrients’ intake. Since the cut-off scores for the identification of the risk category differ between the two sections, analyses were performed separately for each Section. Results from Section 1 and Section 2 follow.

### NutricheQ Questionnaire – Section 1

Depending on the score obtained in Section 1 (Question 1–4), 29 toddlers (14.4%) were included in the low-risk nutritional category (score 0–1), 113 (56.2%) in the moderate-risk category (score 2–3), and 59 (29.3%) in the high-risk category (score 4–8). Considering the median (IQR) intake of each nutrient, derived from the 3DWFR, significant between-group differences were observed in terms of iron, vitamin D, zinc, and proteins intakes (Table 3).

**Table 3:** Intakes of macro- and micronutrients according to the risk groups

	Low-risk group	Moderate-riskgroup	High-risk group	*p*
NutricheQ Section 1				
*Score*	*0–1*	*2–3*	*4–8*	
*N*	*29*	*113*	*59*	
Carbohydrates (g)	131.0 (48.7)	134.9 (39.2)	127.7 (51.1)	0.46
Carbohydrates (%TE)	49.3 (4.6)	47.8 (7.3)	46.5 (7.3)	0.06
Proteins (g)	33.7 (14.8)	44.95 (12.1)	43.97 (17)	<0.00
Proteins (g/kg_BW_)	2.82 (1.4)	3.63 (1.3)	3.09 (1.13)	0.00
% Proteins (%TE)	14.7 (3.6)	16.65 (3.45)	16.98 (3.4)	0.01
Fats (g)	35.6 (9.4)	40.5 ( 12.1)	41.6 (12.3)	0.00
Fats (%TE)	33.3 (4.7)	35 (5.1)	35.6 (5.6)	0.02
Folic acid (µg)	126.85 (84.55)	140.85 (79.92)	118.08 (64.05)	0.03
Vitamin A (µg)	497.82 (258.95)	635.09 (446.84)	512.51 (393.38)	0.06
Vitamin D (µg)	4.4 (3.04)	0.9 (1.15)	0.89 (0.6)	<0.00
Vitamin E (mg)	8.26 (4.58)	6.92 (2.13)	5.88 (1.98)	<0.00
Iron (mg)	7.34 (2.6)	5.79 (2.05)	4.67 (1.9)	<0.00
Calcium (mg)	530.93 (177.23)	646.8 (368.42)	596.24 (207.9)	0.04
Potassium (mg)	111.31 (440.87)	1528.59 (555.74)	1506.93 (595.93)	<0.00
Zinc (mg)	6.37 (3.02)	6.2 (2.55)	5.45 (1.73)	0.00
NutricheQ Section 2				
*Score*	*0–2*	*3–5*	*6–14*	
*N*	*60*	*112*	*29*	
Sugars (g)	54.25 (22.04)	52.56 (22.94)	49.14 (32.35)	0.47
Sugars (% TE)	19.2 (5.63)	19.38 (7.08)	18.14 (8.44)	0.48
Saturated fatty acids (g)	13.1 (7.75)	12.99 (6.43)	11.23 (4.79)	0.24
Saturated fatty acids (% TE)	11.11 (4.29)	11.14 (4.09)	10.55 (3.94)	0.48
Fibre (g)	9.1 (4.7)	7.5 (4.2)	6.2 (2.8)	<0.00
Fibre (g/4184 kJ)	8.5 (4.5)	7 (3.9)	5.6 (2.45)	<0.00
Thiamine (mg)	0.62 (0.18)	0.6 (0.22)	0.5 (0.17)	0.03
Riboflavin (mg)	1.21 (0.46)	1.14 (0.5)	0.95 (0.3)	0.02
Niacin (mg)	7.44 (3.29)	7.82 (4.21)	7.43 (4.06)	0.70
Folic acid (µg)	148.27 (74.93)	129.63 (81.72)	110.36 (43.39)	0.00
Vitamin C (mg)	84.8 (50.2)	59.4 (52.8)	47 (40)	<0.00
Sodium (mg)	714.58 (468.24)	637.04 (332.42)	593.2 (312.4)	0.23
Potassium (mg)	1571 (607)	1491 (610)	1194 (577)	<0.00
Calcium (mg)	649 (319)	608 (297)	432 (258)	0.00
Phosphorus (mg)	812 (283)	759 (230)	589 (267)	0.01
Fruit (g)	131 (109.6)	100 (112)	50 (93)	<0.00
Vegetables (g)	33 (67)	20 (50)	67 (33)	0.06

Intakes of macro- and micronutrients in low-, moderate-, and high-risk groups, as categorised by NutricheQ Sections 1 and 2, are reported as median (IQR). Intakes were assessed using a 3-day weighed food record. The significant differences in nutrient intakes in between-groups are reported as p-values.

According to the ROC analysis performed, a score of ≥4 identified toddlers with a poor iron intake (AUC=0.678; *p*=0.001); thus, toddlers included in the high-risk group category (score 4–8) are at possible risk of poor iron intake; on the contrary, a score of ≥2 identified toddlers exceeding recommended%TE protein intake (AUC=0.6024; *p*=0.009). Thus, toddlers included in the high-risk category reasonably consume an excessive amount of proteins, as well as those in the medium-risk group.

None of the toddlers met the LARN recommendation for vitamin D intake, thus the ROC analysis was not performed.

To rule out whether the SES affected the child's risk status, the percentage of children with mothers who hold a doctoral degree (low risk=50%; moderate risk=47%, and high risk=53%; p=ns) or the percentage with at least one parent who has a doctorate (low risk=58%, moderate risk=59%, and high risk=63% p=ns) were compared among groups. The figures did not differ significantly.

The prevalence of obese parents among risk group categories was compared as well, but no significant difference was observed.

### NutricheQ Questionnaire – Section 2

Depending on the scores obtained in Section 2 (Question 5–11), 60 toddlers (29.9%) were included in the low-risk nutritional category for dietary imbalances (score 0–2), 112 (55.7%) in the medium-risk category (score 3–5), and 29 (14.4%) at high risk (score 6–14). Table 3 reports the 3DWFR median (IQR) consumption of carbohydrates, fats, fibre, thiamine, riboflavin, niacin, folic acid, vitamin C, sodium, potassium, fruits, and vegetables for each group of risk, as well as between-group statistical differences. Toddlers included in the low-risk group showed an adequate intake of fruit (160 g/day); on the contrary, those included in the high-risk group did not meet recommendations, with a median consumption of 50 g of fruits per day (Table 3). Taking into consideration fibre intake, a score of ≥3 identified, with good accuracy, toddlers with poor intake (AUC=0.7028; *p*<0.0001).

As observed in Section 1, there were no SES differences between the risk groups (the prevalence of mothers holding a doctoral degree was 54% for toddlers at low risk, 52% for those at moderate risk, and 33% for high-risk toddlers; p=ns. The prevalence if at least one parent has a doctorate was 66% in at low-risk toddlers; 59% at moderate risk, and 53% at high risk; p=ns). No difference was found in the prevalence of parents with obesity class I to III.

### Test–retest reliability

To assess the reliability of the tool, NutricheQ was administered twice to an additional sample (validation set) of 50 subjects within a range time of 10 to 15 days. The NutricheQ overall score was reliable between administrations. Supplementary Table 4 reports the percentage of toddlers belonging to the different risk classes at each administration for both questionnaire sections. For Section 1, the ICC was 0.73 (95% CI 0.40-0.89; F=6.3; *p*=0.0002); the Cronbach's alpha 0.83. For Section 2, the ICC was 0.55 (95% CI 0.13-0.81; F=3.5; *p*=0.0074); and the Cronbach's alpha 0.70.

### Nutrient intakes from 3DWFR

Finally, energy and macro- and micronutrients intake of the whole sample (training set) were assessed to provide a generic overview of the overall population recruited. Results are summarised in Table 2, as well as Italian DRVs for infants. Our findings show that intakes of fibre, folic acid, vitamin D, potassium, iron, and calcium were below the LARN recommendations, whereas the intake of sugars exceeded the suggested dietary target (SDT).

Eleven males (10.2%) and eight girls (8.6%) exceeded the LARN recommended TE intake. In the whole sample, five toddlers (2.5%) exceeded the LARN recommended values for carbohydrates (%total energy; TE) and 28 (13.9%) the DRVs for fats (%TE); many toddlers had an excessive consumption of refined sugars (*n*=159; 79.1%) and saturated fatty acids (*n*=124; 61.7%). Regarding proteins, 140 toddlers (69.7%) had an intake (%TE) above 15%. Finally, 129 toddlers (64.2%) did not reach the recommendations for daily intake of fibre.

The percentage and number of toddlers who did not meet the recommended intake of vitamins and minerals is reported in Table 2b and c.

Our survey was not intended to explore the consumption of cows’ milk or foods other than milk and dairy products that can cause reduced iron intake and high protein intake. Nevertheless, we also focused on cow's milk consumption in our sample. According to the 3DWFR, 142 out of 201 toddlers (70.7%) consumed cow's milk. The average daily consumption was 288 ml (about two glasses), with a minimum and a maximum value of 30 ml and 693 ml, respectively. We observed that the percentage of toddlers consuming cow's milk increased from the low-risk to the high-risk group in Section 1: 10.3%, 78.8%, and 88.1% of toddlers consumed cow's milk in the low-risk, moderate-risk, and high-risk groups, respectively.

## Discussion

The NutricheQ Questionnaire is a reliable tool that identifies those toddlers with high probability of having too low or excessive intake of some specific macro- and micronutrients, due to dietary imbalances and less than ideal eating habits. Given the questionnaires currently available, the NutriSTEP Questionnaire has been developed to assess dietary risk in preschoolers (16), while others have been recently designed and validated for toddlers aged 12 to 36 months, such as the TDQ (17). Both NutricheQ and TDQ identify risk categories (three and four, respectively) depending on the score obtained in each section. Unlike the NutricheQ Questionnaire, the TDQ validity was assessed through the correlation between its risk categories scores and the dietary risk scores derived from a food frequency questionnaire. Instead, the risk categories of our tool were compared to an exhaustive nutritional assessment, obtained from a 3DWFR that reflected the detailed food consumption of the toddlers involved in the study. Despite TDQ being useful in the identification of dietary risk, described as ‘any appropriate dietary pattern that may impair health’, NutricheQ focuses on some specific aspects of dietary imbalances, primarily those dietary patterns and habits that might lead to poor iron and vitamin D intake, a matter of serious concern for Italian toddlers, as described in the Nutrintake 636 Study (1).

The tool is short and easy to complete by parents and caregivers, providing the paediatrician with consistent information, as demonstrated by the test–retest statistics, on the toddler's intake of macro- and micronutrients. Moreover, scores within each section are rapidly computable: the tool can be administered during the periodic visits for growth monitoring, to identify those toddlers who require blood screening for the deficiency of some micronutrients and/or nutritional intervention.

### Section 1

Items in Section 1 concern milk consumption, breakfast products, meat, and fish. It is informative about the intake of iron and vitamin D, proteins, and fats.

In our survey, toddlers belonging to the high-risk group as identified in Section 1 showed a poor intake of iron and an excessive intake of proteins and fats. The intake of iron was below the recommended dietary allowance (4 mg/day, according to LARN recommendations) in most of the toddlers, (23) and the intake of vitamin D was below recommendations in all of them.

The inadequate intake of iron in toddlers is of serious concern in westernised countries, where iron-deficiency anaemia and iron deficiency without anaemia during infancy and early childhood are highly prevalent (24). Because of their rapid growth and brain development, infants and toddlers need more iron than older children and adults. Iron deficiency may impair neurodevelopment, affecting in particular verbal learning and memory capacities (9, 10). The American Academy of Paediatrics, indeed, recommends universal screening for anaemia by determining haemoglobin concentration at approximately 1 year of age in all the toddlers (25). To reduce the burden of iron deficiency at this age, the European Society for Paediatric Gastroenterology, Hepatology and Nutrition (ESPGHAN) Committee on Nutrition has recently recommended that toddlers should receive iron-rich complementary foods, including meat and iron-fortified foods, and should avoid the excessive consumption of unmodified cow's milk (26). Other nutritional strategies include avoiding the consumption of foods containing phytates and enhancing the consumption of food rich in vitamin C.

In this context, the NutricheQ may help paediatricians and health care providers to identify toddlers at risk of presenting iron deficiency who may need blood screening and even nutritional intervention.

Interestingly, in our survey, toddlers who were at high risk for iron deficiency had an excessive intake of proteins. Epidemiological studies suggest an association between iron deficiency and excessive intake (e.g. 600 ml/day) of cow's milk. The latter would prevalently not meet all the nutritional requirements of the growing child (this is the case with iron) and would cause an excessive protein intake (27, 28).

For vitamin D, the LARN recommendations set a dietary reference value of 10 µg/day. This is in line with the adequate intake set by the Scientific Committee for Food (SCF) (based on serum 25(OH) D concentrations) and considered by EFSA to be adequate for the majority of young toddlers having minimal sun exposure (5). None of the subjects included in our sample met the recommendations set for toddlers. Since the aim was to estimate the risk of possible nutrient imbalances related to diet, supplementation with vitamins or minerals was not investigated. In any case, significant differences were found between the three risk groups, showing that particular attention should be paid to the vitamin D intake of toddlers in the high-risk group.

In our sample, toddlers at high risk also showed an excessive consumption of proteins (more than 15% of TE intake derived from proteins) and fats that can boost their risk of developing obesity and cardiometabolic abnormalities in childhood and early adulthood. These findings are in keeping with evidence from the literature that describes how in the period from one through 3 years of age, there is a marked increase in proteins intake from the 5% of Energy from Protein (PE%) observed in infants who have been breastfed exclusively to the 15 PE% observed in infants who have been introduced to complementary foods. At this age, the mean proteins intake is ~3 times higher than the physiologic requirement, but some toddlers receive four to five times their physiologic requirement (2, 6).

High protein intake by infants between 6 and 24 months old stimulates the IGF-1 axis and insulin release (29), anticipating the time of the adiposity rebound (30). Protein from cow's milk constitutes a main part of protein intake in toddlers, and it seems to have a specific effect on IGF-1 concentration and growth (6). The FITS study has demonstrated that patterns of excessive protein intake are set as early as 18 months of age; by 20 months of age, the pattern of US toddlers mimic that of adults (2).

### Section 2

Items in Section 2 concern the intake of fruit, fibre, and microelements, including non-liposoluble vitamins. The intake of fibre and fruits decreased from the low- to the high-risk category, as well as the intake of vitamin C.

Our findings are in keeping with recent evidence from the Nutrintake 636 Study, an epidemiological study investigating the nutritional habits of 636 Italian toddlers aged <12 months using a 7-day weighed food record. Despite having normal anthropometry and energy intake, toddlers had a high intake of proteins, sugars, saturated fatty acids, and sodium; and a low intake of iron and fibre compared to Italian DRVs (1). Recently, the inadequate intake of fruits and vegetables has been reported also in Dutch toddlers (3).

Fruits and dietary fibre are an important source of micronutrients, water, and chemicals; and they exert beneficial effects on a child's health and well-being. It is widely accepted that consuming fruits and vegetables increases satiety and satiation, protecting against excessive energy intake, and hence overweight and obesity (31). Adequate intake of fibre for Italian toddlers older than 1 year of age consists of 14 portions of fruit and 14 portions of vegetables per week (31). The median daily consumption of fruit and vegetables in our sample was 100 g and 20 g, respectively. One serving consists of about 100 g of pureed fruits (approximately 80 g of fresh fruit) and 40 g of pureed vegetables (approximately 15 g of fresh vegetables, excluding potatoes).

In toddlers younger than 2 years of age, the low intake of dietary fibre has been associated with an enhanced risk of constipation and appendicitis in childhood (33, 34).

Western societies generally consume vitamin C (≥10 mg a day) that is more than sufficient to prevent scurvy. In toddlers, a suboptimal intake of vitamin C, as seen in the high-risk group, may contribute to iron deficiency. In our sample, the median intake of vitamin C was similar to values recently found in German toddlers, as reported in a large-scale, cross-sectional study investigating toddlers’ diet (35).

## Limitations

Our study was not exempt from limitations (approximate records of dietary intakes, imprecise food composition tables, and the failure to take into account the bioavailability of nutrients in relation to diet and physiological status), although we found these flaws to be in common with several food consumption surveys. Longitudinal studies with recurrent blood sampling, which would be the gold standard, are very hard to carry out due to ethical considerations, high cost, and a high likelihood of poor compliance by participants. A 3DWFR has been used to evaluate the changes of the nutrient intake of infants involved in clinical studies (36, 37), even though this tool may be not as accurate as the 7DWR in computing nutrient intake (38).

For the test–retest, the NutricheQ was completed twice within an interval of time ranging from 10 to 15 days. The interval between the test and retest was intentionally short because, in the early stages of life, both the quality and quantity of food consumption suddenly changes. However, we cannot rule out that such a short time frame could skew the study's results, since parents may remember the answers they gave in the first administration of the questionnaire.

Due to the high educational and employment levels reported, it may seem that economically privileged and well-educated families were selected for the study, but that was not the case. The questionnaire was developed to be administered without any distinction to families that range in income and education. Considering that diet and eating habits are socially patterned, the reliability and validity of the NutricheQ Questionnaire for low-income and less educated families are yet to be assessed.

## Conclusions

The NutricheQ Questionnaire seems to be a reliable tool for assessing the dietary risk factors in toddlers. NutricheQ focuses on some specific aspects of dietary imbalances, primarily those dietary patterns and habits that might lead to poor iron and vitamin D intake, and could be easily applied in daily clinical practice.

## Supplementary Material

NutricheQ Questionnaire assesses the risk of dietary imbalances in toddlers from 1 through 3 years of age
