# Supplementary material for: NutricheQ Questionnaire assesses the risk of dietary imbalances in toddlers from 1 through 3 years of age
Source: Food Nutr Res. 2015 Dec 18;59:10.3402/fnr.v59.29686. doi: 10.3402/fnr.v59.29686 (PMC4685971; doi:10.3402/fnr.v59.29686)
Supplement: NutricheQ Questionnaire assesses the risk of dietary imbalances in toddlers from 1 through 3 years of age [file FNR-59-29686-s001.pdf]

Supplemental table 1. Italian version of the NutricheQ questionnaire.

PARTE 1 - QUESTIONARIO ALIMENTARE PER BAMBINI DA 1 A 3 ANNI

A: *Per ciascuna delle seguenti domande, segna con una crocetta (x) la risposta che ritieni più corretta.*

1. A quale età il tuo bambino è passato al latte vaccino come latte principale?	Non è ancora passato al latte vaccino ☐	A 12 mesi o successivamente ☐	Prima dei 12 mesi ☐
2. Il mio bambino beve abitualmente nell'arco di una giornata:	Latte crescita 1 o più volte ☐	fino a 500ml circa di latte vaccino/latte materno ☐	più di 500ml di latte vaccino ☐
3. Il tuo bambino mangia abitualmente carne rossa (manzo, agnello, maiale) o pesce (sardine, tonno, sgombro o salmone)	≥3 giorni la settimana ☐	Una o due volte la settimana ☐	Raramente o mai ☐
4. Per colazione il tuo bambino consuma alimenti arricchiti in ferro (es. cereali/biscotti per l'infanzia)?	Tutti i giorni o quasi ☐	Alcune volte alla settimana ☐	Una volta a settimana o mai ☐

B: *Per ciascuna delle seguenti domande, segna con una crocetta (x) l'opzione che ritieni più corretta.*

5. Evito di dare al mio bambino alcuni alimenti a causa di allergie o intolleranze alimentari *(ad esempio, latte/ cereali con glutine/pesce/uova/soia)*	NO – non ci sono tipologie di alimenti che evito ☐	SI – un tipo di alimento ☐	SI – 2 o più tipi di alimenti ☐
6. Di solito il mio bambino mangia frutta: (*non considerare i succhi di frutta*)	VERO –≥3 volte/porzioni al giorno ☐	ABBASTANZA VERO –1 o 2 volte / porzioni al giorno ☐	RARAMENTE o MAI ☐
7. Il mio bambino mangia verdura:	VERO – ne consuma 2 porzioni tutti i giorni o quasi ☐	ABBASTANZA VERO - ne mangia un po' ma non come dovrebbe o come io vorrei ☐	FALSO – Ne mangia poca o non ne mangia (non gli piace/ne mangia poche varietà o nessuna) ☐
8. Il mio bambino mangia latticini: *(ad esempio formaggio e yogurt)*	2 - 4 volte/porzioni al giorno ☐	Più di 4 volte/porzioni al giorno ☐	< 2 volte/porzioni al giorno ☐
9. Il mio bambino mangia più "cibi pronti" di quanto dovrebbe *(ad esempio patatine fritte, hamburger, wurstel, crocchette di pollo etc...)*	FALSO – al massimo 1-2 volte a settimana ☐	ABBASTANZA VERO – tre/quattro volte la settimana *(es. perché è veloce da preparare, gli piace, lo mangia fuori casa)* ☐	VERO – Quattro o più volte la settimana *(es. perché è veloce da preparare, gli piace, lo mangia fuori casa)* ☐
10. Il mio bambino ottiene più "ricompense" *(cioccolato, caramelle, biscotti, torte, dolci, gelati etc.)* di quanto dovrebbe	FALSO – solo occasionalmente ☐	ABBASTANZA VERO - la maggior parte dei giorni e forse un po' più di quanto dovrebbe ☐	VERO – difficile da trattenere o limitare *(es. è schizzinoso e mangia poco quindi almeno in questo modo mangia qualcosa)* ☐
11. Il mio bambino di solito beve succhi di frutta o altre bevande zuccherate non a base di latte	Mai o meno di una volta al giorno (meno di 150ml) ☐	1-2 volte al giorno (150-200ml) ☐	Più di 2 volte al giorno (più di 200ml) ☐

*Supplemental table 2***.** Italian LARN recommendations and European EFSA Dietary Reference Values (DRVs) or adequate levels (*) for toddlers` nutrients intake.

	LARN				EFSA
Nutrients	**AR**	**AI**	**RI**	**SDT**	
Energy (kJ)	M: 3640.1-5815.8<br>F: 3305.4-5355.5				M: (AR)<br>5355.5- 4912.0<br>F: (AR) 2979.0- 4585.7
Proteins (g)					
Proteins (g/kg $_{BW}$)	0.82				0.83 (AR)
Proteins (%TE)				<15	
Carbohydrates (g)					
Carbohydrates (%TE)			45-60		45-60 (RI)
Sugars (g)					
Sugars (%TE)					
Fats (g)					
Fats (%TE)			35-40		35-40 (RI)
Saturated fatty acids (g)					
Saturated fatty acids (%TE)				<10	
Total fibre (g)*					10
Fibre (g/4184kJ)		8.4			8.39
Thiamine (mg)*	0.3				0.5
Riboflavin (mg)*	0.4				0.8
Niacin (mg NE)	5				M:4.3-6.4 (AR)<br>F:3.9-6 (AR)
Folic acid (µg)	110				60 mcg DFE
Vitamin A (µg RE)	200				205 (AR)
Vitamin C (mg)	25				15 (AR)
Vitamin D (µg)*	10				10
Vitamin E (mg)*		5			6
Sodium (mg)*		700		900	170-370

Potassium (mg)*		1700	800
Iron (mg)*	4		8
Calcium (mg)*	500		600
Phosphorum (mg)*	380		460
Zinc (mg)	4		3.6 (AR)
Selenium (µg)	16		15 (AI)

The LARN reference values are reported as: AR (Average Requirement), AI (Adequate intake), RI (Reference Intake) and SDT (Suggested Dietary Target). The EFSA values are reported as Dietary Reference Values (AR or RI) or adequate levels (*).

*Supplemental table 3***.** Parents' characteristics.

		Fathers (n=201)	Mothers (n=201)
**Education**	Primary	1.1 %	0%
	Upper secondary/post secondary non tertiary	16.9%	9.0%
	Bachelor degree	47.2 %	41.6%
	Doctoral or equivalent	34.8 %	49.4%
**Employment**	Unemployed	2.2%	20.2%
	Employed	59.6%	60.7%
	Freelance	38.2%	19.1%
	Retired	0%	0%
**BMI class**	Under-weight	0%	12.6%
	Normal-weight	47.1%	62.1%
	Over-weight	46.0%	20.7%
	Obesity class I	2.3%	2.3%
	Obesity class II	4.6%	1.1%
	Obesity class III	0%	1.1%

Education levels were categorized according to the International Standard Classification of Education 2011 criteria and classes of body mass index (BMI) according to the WHO criteria**.**

Supplemental table 4. Test-retest reliability**.**

**SECTIONS**	**ADMINISTRATIONS**	**Risk Level**	**n**	**Percentage**
Section 1	Administration 1	Low (L)	4	23.53%
		Moderate (M)	3	17.65%
		High (H)	10	58.82%
	Administration 2	Low (L)	4	23.53%
		Moderate (M)	6	35.3%
		High (H)	7	41.17%
Section 2	Administration 1	Low (L)	2	11.76%
		Moderate (M)	14	82.36%
		High (H)	1	5.88%
	Administration 2	Low (L)	2	11.76%
		Moderate (M)	13	76.48%
		High (H)	2	11.76%

The number and percentage of participants included in the low-, moderate and high-risk groups in the first and second administration of the questionnaire are shown both for Section 1 and Section 2.
